# PIWIL2 interacting with IKK to regulate autophagy and apoptosis in esophageal squamous cell carcinoma

**DOI:** 10.1038/s41418-020-00725-4

**Published:** 2021-01-19

**Authors:** Xu Zhao, Lian Huang, Yilu Lu, Wenhao Jiang, Yue Song, Bojun Qiu, Dachang Tao, Yunqiang Liu, Yongxin Ma

**Affiliations:** Department of Medical Genetics, Frontiers Science Center for Disease-related Molecular Network, State Key Laboratory of Biotherapy, West China Hospital, Sichuan University and Collaborative Innovation Center, Chengdu, 610041 China

**Keywords:** Macroautophagy, Prognostic markers, Cancer genetics

## Abstract

Esophageal squamous cell carcinoma (ESCC) is one of the most common malignancies and cause of death from cancer in China. Previous studies showed that autophagy and apoptosis inhibition are critical for the survival of ESCC cells. However, the underlying mechanisms remain to be clarified. Recently, we found that PIWIL2, a novel cancer testis protein, is highly expressed in ESCC and associated with high T-stage and poor 5-year survival rate in patients. Our further study showed that PIWIL2 can directly bind to IKK and promote its phosphorylation, leading to phosphorylation of IκB and subsequently nuclear translocation of NF-κB for apoptosis inhibition. Meanwhile, PIWIL2 competitively inhibits binding of IKK to TSC1, and thus deactivate mTORC1 pathway which suppresses ULK1 phosphorylation and initiation of autophagy. The mouse xenograft model suggested that PIWIL2 can promote ESCC growth in an IKK-dependent manner. This present work firstly revealed that PIWIL2 can play a role in regulating autophagy and apoptosis, and is associated with poor prognosis in ESCC patients, providing novel insights into the roles of PIWIL2 in tumorigenesis.

## Introduction

Esophageal cancer is the eighth most prevalent cancer type and the sixth-leading cause of cancer-associated mortality worldwide [1]. Esophageal adenocarcinoma (EAC) and esophageal squamous cell carcinoma (ESCC) comprise the two common histological subtypes of esophageal carcinoma. And ESCC is more prevalent in East Asia like China (>90%), while EAC occurs most frequently in Western nations [2]. ESCC displays low 5-year survival rate (<30%) that are associated with late stage diagnosis, frequent metastasis, and therapeutic resistance [3, 4]. In recent years, autophagy inhibitors have been reported to overcome multidrug resistance of cancer cells, suggesting that autophagy is a new target for ESCC therapy [5–8].

Autophagy is a highly conserved catabolic process through which cellular constituents like dysfunctional proteins and damaged organelles are sequestered by autophagic vacuoles (AVs), and then delivered to lysosomes for hydrolytic degradation [9]. One well-established negative regulator of autophagy is mammalian target of rapamycin complex 1 (mTORC1) that act to modulate ULK1-mediated nucleation of AVs. The subsequent molecular mechanism of autophagy involves several conserved autophagy-related (ATG) proteins. LC3-II (ATG8) is generally considered as a marker for autophagy, and Beclin-1 (ATG6) is one of the positive regulators of autophagy. However, previous studies indicated that autophagy may promote esophageal cell survival or cell death in a context-dependent manner [10–13], leaving the underlying mechanism of autophagy regulation in ESCC largely unknown.

In addition to mTORC1, the nuclear factor kappa B (NF-κB) signaling pathway has been implicated in autophagy [14]. The IκB kinase (IKK) complex phosphorylates the inhibitory molecule IκB, resulting in ubiquitination and degradation of IκB. Subsequently, released NF-κB dimers translocate to the nucleus and bind to κB sites on target genes involving apoptosis inhibition and cell survival [15]. Notably, recent work has revealed that IKKβ can directly interact with and phosphorylate tuberous sclerosis 1 (TSC1) to active mTORC1, thus regulate autophagy in an NF-κB-independent manner [16].

*PIWIL2* (piwi-like RNA-mediated gene silencing 2), aka HILI in human, is a member of PIWI family defined by highly conserved PAZ and PIWI domains [17]. PIWIL2 is predominantly expressed in testis of normal adults, but also widely expressed in various tumors [18–20]. Our previous researches had showed that PIWIL2 plays roles in tumorigenesis and tumor development through several underlying mechanisms. PIWIL2 promoted cancer cell proliferation via increasing c-Myc expression by facilitating NME2 binding to the G4-motif [21], facilitated cancer cell migration via TBCB [22] and resisted apoptosis by regulating TP53 and TGF-β receptor [23–25].

In this study we present that PIWIL2 can directly interact with IKK, the major regulator of the canonical NF-κB pathway, and enhance IKK phosphorylation. PIWIL2-induced IKK activation promotes NF-κB nuclear translocation and suppresses apoptosis in ESCC cells. Meanwhile, PIWIL2 competitively blocks the interaction of IKK with TSC1, thus inhibiting mTORC1 signaling and promoting autophagy. Tissue microarray (TMA) and bioinformatics analysis indicated that PIWIL2 is highly expressed and associates with poor prognosis in ESCC patients. These results revealed the molecular mechanism that PIWIL2 can suppress apoptosis and promote autophagy in ESCC, providing a novel insight into roles of PIWIL2 in tumorigenesis.

## Results

### PIWIL2 is highly expressed and associated with T-stage in ESCC

**Fig. 1:**
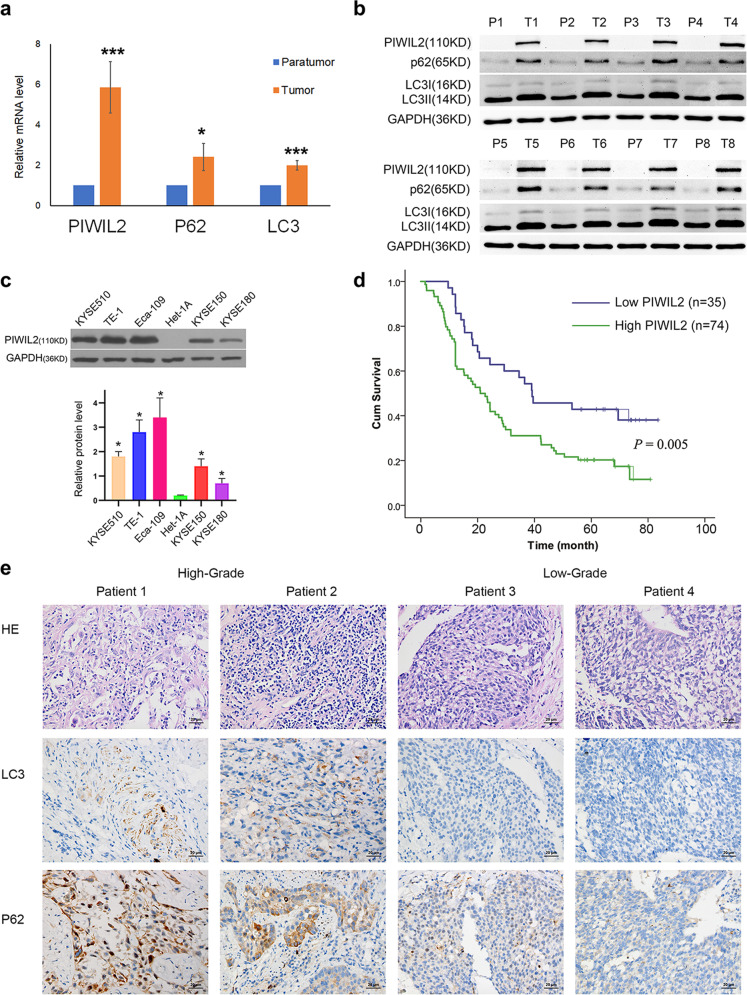
PIWIL2 is highly expressed and associated with poor prognosis in ESCC. Total mRNAs and proteins were extracted from tissue samples of eight individual ESCC patients, and subjected to RT-PCR (**a**) and western blot (**b**) respectively. T, tumor tissues; P, paratumor tissues. **c** ESCC cell lines (KYSE150, KYSE180, KYSE510, and Eca-109) and normal esophageal epithelial cell line Het-1A were subjected to expression analysis of PIWIL2 using western blotting (WB). **d** Kaplan–Meier curve depicting the long-term survival of the ESCC patients (*n* = 109). The curves were stratified based on the PIWIL2 level scored by intensity (0–3) and area (0–4) of the staining with TMA and IH technology (Log-rank test, *p* = 0.005). **e** Patients were randomly selected from PIWIL2 high-expression group and low-expression group (two from each). Biomarkers of autophagy (LC3 and P62) were analyzed using immunohistochemistry. Scale bar, 20 µm. *p < 0.05; ***p < 0.001.

To examine the expression of *PIWIL2*, matched paratumor tissues and ESCC tissues from eight unrelated patients were subjected to RT-qPCR and western blot (WB) analysis. In the paratumor tissues, PIWIL2 expression can not be observed at protein level; while all carcinoma tissues showed notable expression of PIWIL2 mRNA and protein (Fig. 1a, b). Also, the expression level of p62 and LC3-II in esophageal carcinoma were significantly higher than those in corresponding paratumor tissues, suggesting high level of autophagy in ESCC. We further studied the expression level of PIWIL2 in five ESCC cell lines, including KYSE150, KYSE510, KYSE180, TE-1, and Eca-109 using WB. Immunostaining can be observed in all ESCC cell lines, but not in immortalized normal esophageal epithelial cell line Het-1A (Fig. 1c).

To reveal the correlation between PIWIL2 and clinicopathological indexes and prognosis of ESCC, TMAs consist of 109 ESCC patients were subjected to immunohistochemistry (IHC) and the expression for PIWIL2 was scored by multiplying intensity value (0–3) and percentage of staining area (0–4). The final scores of all samples were analyzed using X-tile software [26] (Supplementary Fig. S1), and divided into low- (score 0–4, *n* = 35) and high-PIWIL2 expression group (score 6–12, *n* = 74). Kaplan–Meier cumulative survival curves with a log-rank test showed that long-term survival rate was significantly lower in patients with high-PIWIL2 expression (*p* = 0.005; Fig. 1d).

Univariate analysis showed that T-stage distribution were significantly associated with PIWIL2 expression (*p* = 0.02). However, no significant association was found between PIWIL2 expression and patients’ age, gender, degree of differentiation, N-stage, or TNM-stage (Table 1). In addition, Cox multivariate analysis showed that PIWIL2 expression level was an independent prognostic factors of patients with ESCC (Hazard ratio 1.81, 95% CI 1.09–3.00, *p* = 0.02; Table 2).

**Table 1:** Relationship between PIWIL2 protein expression and clinical pathological indexes.

	PIWIL2		*p* value
	Low expression (%)	High expression (%)	
Gender			0.58
Female	4 (11.4)	12 (16.2)	
Male	31 (88.6)	62 (83.8)	
Age			0.31
<60 years	21 (60)	36 (48.6)	
≥60 years	14 (40)	38 (51.4)	
Differentiation			0.32
G1	1 (2.9)	8 (10.8)	
G2	24 (68.6)	43 (58.1)	
G3	10 (28.6)	23 (31.1)	
Position			0.90
Upper	15 (42.9)	29 (39.2)	
Middle	14 (40)	33 (44.6)	
Lower	6 (17.1)	12 (16.2)	
Size (cm)			0.35
≤3	8 (10.8)	13 (11.9)	
3–5	22 (29.7)	32 (29.4)	
≥5	44 (59.5)	64 (58.7)	
pT			0.02
T1	4 (11.4)	0 (0)	
T2	8 (22.9)	16 (21.6)	
T3	19 (54.3)	43 (58.1)	
T4	4 (11.4)	15 (20.3)	
pN			0.32
N0	21 (60)	33 (44.6)	
N1	9 (25.7)	20 (27)	
N2	3 (8.6)	16 (21.6)	
N3	2 (5.7)	5 (6.8)	
TNM-stage			0.05
I	3 (8.6)	0 (0)	
II	18 (51.4)	33 (44.6)	
III	11 (31.4)	31 (41.9)	
IV	3 (8.6)	10 (13.5)

**Table 2:** Cox multivariate analysis of PIWIL2 and various clinicopathological indexes.

	*p* value	Hazard ratio	95% CI
pT	0.06	1.52	[0.98–2.36]
pN	0.31	1.37	[0.74–2.53]
TNM-stage	0.76	0.86	[0.33–2.23]
PIWIL2	0.02	1.81	[1.09–3.00]

To explore the association between PIWIL2 expression and autophagy, a limited set of tissue samples (two from each group) were subjected to IHC analysis. And the staining of autophagic marker protein p62 and LC3 were notably higher in high-PIWIL2 expression group.

In general, our results suggested that highly expressed PIWIL2 in ESCC is associated with pathological T-stage and may serve as a novel biomarker for prognosis prediction.

### PIWIL2 promotes cell proliferation, inhibits apoptosis, and activates autophagy

**Fig. 2:**
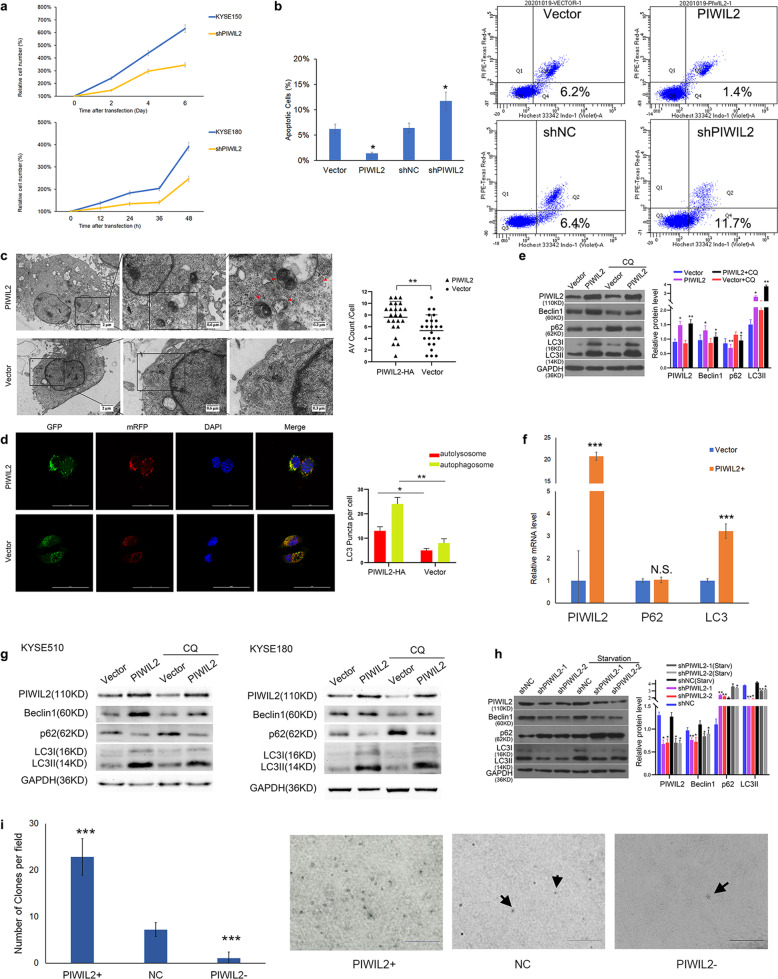
PIWIL2 inhibited apoptosis, promoted cell proliferation, and activated autophagy. **a** Proliferation rates of PIWIL2 knockdown stable KYSE150 cells (upper shPIWIL2) and transient transfected KYSE180 cells (lower shPIWIL2) were revealed by crystal violet assay. Mock-transfected cells were used as control. **b** At 24 h after transfection, KYSE150 cells were treated with serum starvation for 6 h. Starvation induced apoptosis was analyzed by flow cytometry with Hochest33342/PI double staining. Apoptosis(%) = percentage of Hochest+ve/PI-ve cells. **c** Electron microscopy images presenting ultrastructure in representative control (transfected with empty vector) and PIWIL2 overexpressed KYSE150 cells. Arrows show autophagic vacuoles (AV). The number of AV of 24 cells was counted in each section. **d** Control and PIWIL2 overexpressed KYSE150 cells were co-tranfected with Ad-mRFP-GFP-LC3B. The number of autolysosomes (red puncta) and autophagosomes (yellow puncta) of 24 cells was counted in each field. Scale bar, 50 µm. **e** KYSE150 cells were treated with 10 µM chloroquine (CQ) which inhibits the degradation of AV. LC3-II accumulation was measured using WB. **f** KYSE150 cells were subjected to RT-PCR at 48 h after transfection with PIWIL2 overexpression vectors (PIWIL2+) or empty vectors (Vector). Relative mRNA levels were normalized with GAPDH as internal control. **g** KYSE510 and KYSE180 cells were treated with 10 µM chloroquine (CQ) and subjected to WB analysis. **h** KYSE150 cells were starved to induce autophagy after transfected with shRNA against Piwil2 or control. Biomarkers of autophagy (LC3 and P62) were analyzed using WB. **i** Representative images of clone formation in soft agar for KYSE150 cells after transient transfection with PIWIL2 overexpression vector (PIWIL2+), PIWIL2 shRNA vector (PIWIL2−), and empty vector (NC). Scale bar, 0.5 mm. N.S., no significance; *p < 0.05; **p < 0.01; ***p < 0.001.

To further investigate the role of PIWIL2 in ESCC, we constructed a *PIWIL2* knockdown stable cell line derived from KYSE150 cells. Proliferation rates were revealed by crystal violet assay, and the results indicated that PIWIL2 knockdown can significantly suppress the proliferation of ESCC cells (Fig. 2a). Flow cytometry analysis with Hochest33342/PI double staining showed that PIWIL2 significantly suppress the apoptosis of KYSE150 (Fig. 2b) and KYSE180 (Fig. S2) cells.

Meanwhile, transmission electron microscopy was employed to observe the ultrastructure of KYSE150 cells. AVs were counted for 24 randomly selected cells from each section. The result revealed that PIWIL2 overexpression significantly increased the number of AVs from 5.3 ± 2.7 to 7.7 ± 2.6 per cellular cross section (*p* = 0.003, Fig. 2c), suggesting that PIWIL2 can promote autophagy in ESCC cells. To determine the effects of PIWIL2 on autophagosomes and autolysosomes formation, KYSE150 cells were transfected with tandem mRFP-GFP-LC3 adenovirus vectors. Confocal images showed that both yellow and red puncta were significantly increased in PIWIL2 overexpressed KYSE150 cells, indicating an increase in the formation of both autophagosomes and autolysosomes (Fig. 2d). WB results showed that PIWIL2 overexpression can increase the expression level of Beclin-1 and LC3-II, while decrease the level of p62 in ESCC cell lines including KYSE150, KYSE510, and KYSE180. Treatment with 10 µM lysosomal inhibitor chloroquine can further increase LC3-II level in PIWIL2 overexpressed cells, suggesting that PIWIL2 can induce autophagy rather than inhibit autophagy flux in ESCC cells (Fig. 2e, g). RT-PCR quantification showed that PIWIL2 overexpression can increase mRNA level of LC3B, whereas no significant change was observed in P62 mRNA level (Fig. 2f), suggesting that PIWIL2 downregulates P62 by promoting its degradation.

In accord to these findings, starvation induced autophagy can be suppressed by PIWIL2 knockdown in KYSE150 cells (Fig. 2h). Colony formation in soft agar was also performed for KYSE150 cells, the colony number was dramatically increased in PIWIL2 overexpressed cells (from 7.2 ± 0.5 to 22.9 ± 1.3 colonies per field of view, *p* < 0.001) and decreased in PIWIL2 knockdown cells (to 1.1 ± 0.4 colonies per field of view, *p* < 0.001; Fig. 2i).

### PIWIL2 interacts with IKK and affects the phosphorylation of IKK

**Fig. 3:**
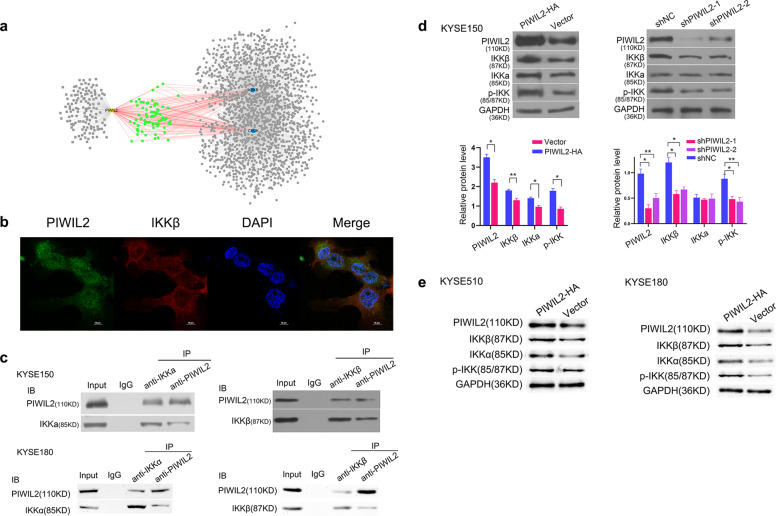
PIWIL2 interacts with IKKβ and affects the phosphorylation of IKK. **a** Protein–protein interaction prediction using PrePPI database revealed that 79 out of 219 proteins in PIWIL2 data set can interact with both IKKα (CHUK) and IKKβ (IKBKB). **b** Immunofluorescence confocal microscopy for localization of PIWIL2 (green) and IKKβ (red) in KYSE150 cells. The nuclei were stained with 1 μg/ml DAPI (blue). Scale bar, 10 µm. **c** Co-immunoprecipitation of PIWIL2 and IKKα/β was performed using indicated antibodies in KYSE150 (upper) and KYSE180 (lower) cells. **d** KYSE150 cells were harvested 48 h after transfection with PIWIL2 expression plasmid (PIWIL2-HA), PIWIL2 shRNA plasmid (shPIWIL2-1/2), or corresponding empty vectors. The lysis were immunoblotted with indicated antibodies and quantified using Image J software. **e** PIWIL2 overexpression upregulated IKK protein level as well as phosphorylation level in KYSE510 and KYSE180 cells. *p < 0.05; **p < 0.01.

To investigate the mechanism of PIWIL2 participating in autophagy and apoptosis, protein–protein interaction (PPI) prediction were performed by using PrePPI database, and the data set was matched with apoptosis and autophagy regulating protein sets. Interestingly, 36% (79/219) of PIWIL2-interacting proteins also interact with IKKα and IKKβ (Fig. 3a). This finding was further supported by using PDB database to predict the potential binding mode, identifying Arg53, Ser60, Asp61, Lys73, and Tyr75 from PIWIL2 and Glu64, Arg140, Asp145, Phe182, and Leu186 from IKKβ as interface residues (Fig. S3).

Also, immunofluorescence localization by confocal microscopy reveals that PIWIL2 and IKKβ were co-localized mainly in the cytoplasm of KYSE150 cells (Fig. 3b). And co-immunoprecipitation assays provide evidence that PIWIL2 can bind with IKKα and IKKβ in KYSE150 and KYSE180 cells (Fig. 3c). In addition, PIWIL2 overexpression in ESCC cells can significantly upregulate both total protein and phosphorylation level of IKKα/β (Fig. 3d, e).

### PIWIL2 activates NF-κB signaling pathway via IKK to regulate apoptosis

**Fig. 4:**
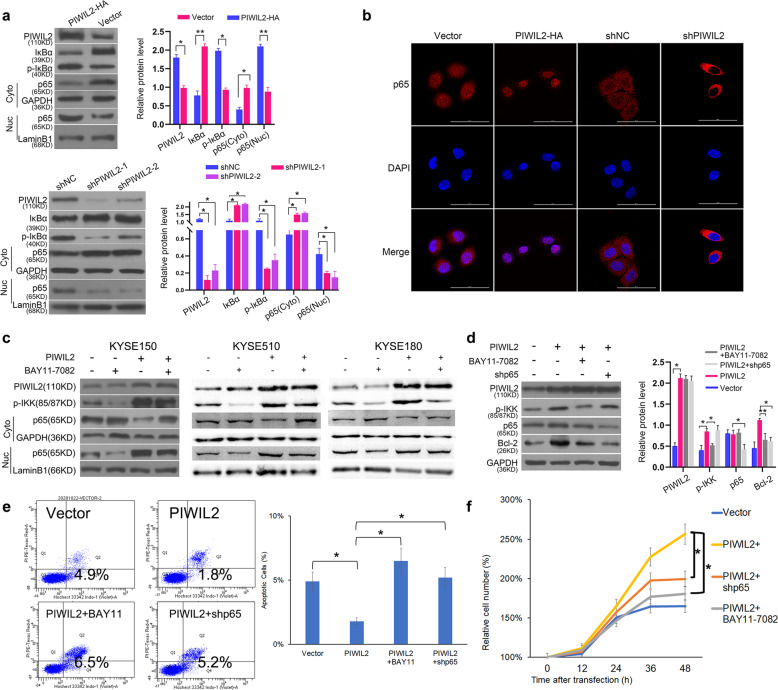
PIWIL2 activates NF-κB signaling pathway through IKKβ to regulate apoptosis. **a** KYSE150 cells were harvested 48 h after transfection and fractionated into nuclear (Nuc) and cytoplasmic (Cyto) components. The translocation of NF-κB were analyzed by measuring p65 subunit level in each component using WB. GAPDH and LaminB1 were employed as internal control, respectively. **b** Immunofluorescence microscopy for localization of p65 in KYSE150 cells 48 h after transfection. The nuclei were stained with 1 μg/ml DAPI (blue). Scale bar, 50 µm. **c** Transfected cells were treated with 10 μM IKK inhibitor BAY11-7082 for 12 h before nuclear/cytoplasmic fractionation. **d** KYSE150 cells with IKK knockdown were treated with BAY11-7082. Autophagic markers (LC3-II and p62) were examined to evaluate the selectively inhibition of BAY11-7082 on IKK. **e** Starvation induced apoptosis was analyzed by flow cytometry with Hochest33342/PI double staining. Apoptosis(%) = percentage of Hochest+ve/PI−ve cells. **f** Proliferation rates of transient transfected KYSE150 cells were revealed by crystal violet assay. Mock-transfected cells were used as control. *p < 0.05; **p < 0.01.

As IKK plays a critical role in phosphorylation of IκB and subsequently activation of NF-κB, we would like to know whether PIWIL2 regulates NF-κB via promoting IKK phosphorylation. In PIWIL2 overexpressed KYSE150 cells, the protein level of IκBα was significantly decreased while the phosphorylation level increased. Also, the nuclear translocation of NF-κB were analyzed by measuring p65 subunit level in fractionated nuclear and cytoplasmic components. WB results showed that overexpression of PIWIL2 can significantly increase p65 level in the nuclear component and decrease p65 level in the cytoplasmic component, suggesting that PIWIL2 promotes NF-κB nuclear translocation (Fig. 4a). Consistently, immunofluorescence results indicated that nearly all p65 staining was present in the cytosol of PIWIL2 silenced KYSE150 cells, while the majority of p65 translocated from the cytosol into the nucleus in PIWIL2 overexpressed cells (Fig. 4b).

In ESCC cells treated with 10 μM IKK inhibitor BAY11-7082, NF-κB nuclear translocation induced by PIWIL2 overexpression was suppressed, suggesting that PIWIL2 activates NF-κB in an IKK-dependent manner (Fig. 4c). Examination of apoptosis marker Bcl-2 also showed that PIWIL2 can suppress apoptosis via IKK/IκB/NF-κB pathway (Fig. 4d). Additionally, Cell Counting Kit-8 and apoptosis analysis showed that PIWIL2 can repress apoptosis and promote cell proliferation by activating IKK/IκB/NF-κB pathway in KYSE150 cells (Fig. 4e, f).

### PIWIL2 inhibits mTOR phosphorylation to regulate autophagy via IKK

**Fig. 5:**
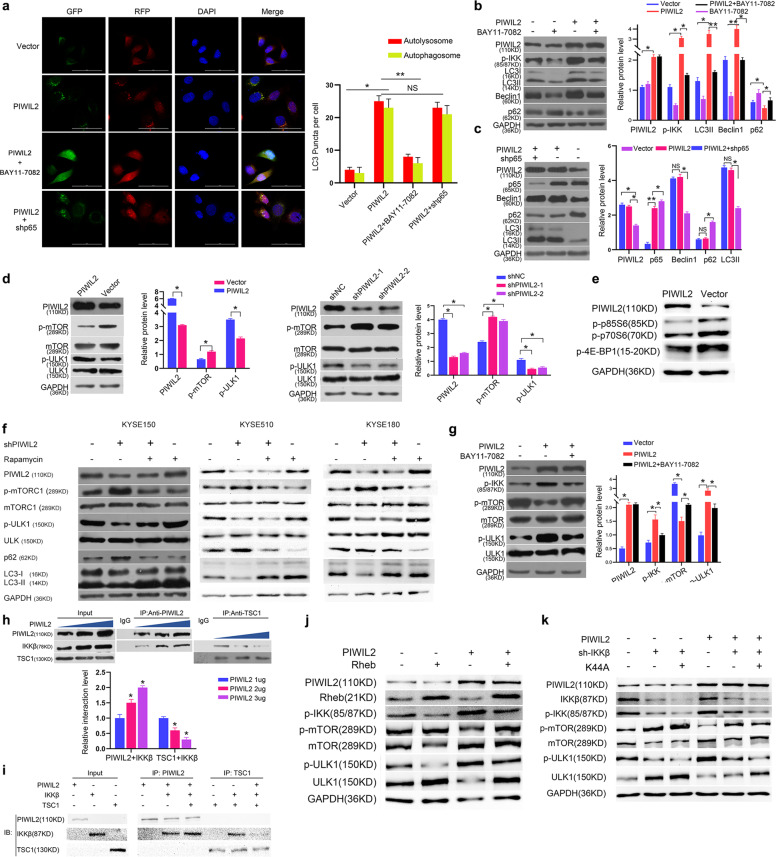
PIWIL2 inhibits mTORC1 phosphorylation to regulate autophagy through IKKβ. **a**–**c** PIWIL2 overexpressed KYSE150 cells were treated with 10 μM IKK inhibitor BAY11-7082 for 12 h or co-transfected with p65 shRNA. **a** Autophagic flux were measured using RFP-GFP-LC3 method. **b**, **c** Protein level of autophagic markers (Beclin-1, p62, and LC3-II) were examined using WB. **d** KYSE150 cells were transfected with PIWIL2 expressed plasmid or empty vector. Phosphorylation level of mTORC1 and ULK1 were examined as markers of mTOR pathway. **e** Phosphorylation level of mTORC1 downstream targets (p85S6, p70S6, and 4E-BP1) were analyzed using WB in PIWIL2 overexpressed KYSE150 cells. **f** Autophage suppressed by PIWIL2 knockdown can be rescued by treatment of 100 nM rapamycin for 12 h to suppress mTORC1 activity in ESCC cell line KYSE150, KYSE510, and KYSE180. **g** Phosphorylation level of mTORC1 decreased by PIWIL2 overexpression can be rescued by BAY11-7082 treatment. **h** KYSE150 cells were transfected with different amount of PIWIL2 vector (1–3 µg). Immunoprecipitation of IKKβ by anti-PIWIL2 or anti-TSC1 was performed to determine the competitiveness of interaction. **i** Cell-free expression of PIWIL2, IKKβ, and TSC1 were conducted using the TnT System, followed with immunoprecipitation analysis. **j** The mTORC1 pathway suppressed by PIWIL2 overexpression can be rescued by co-transfection with Rheb. **k** IKKβ K44A (Kinase-dead variant) was constructed and transfected into KYSE150 cells to rescue knockdown of IKKβ by transfecting shRNA against IKKβ (sh-IKKβ). *p < 0.05; **p < 0.01.

Previous study has reported that IKK contributes to induce autophagy in an NF-κB-independent manner [14]. So we examined whether PIWIL2 induces autophagy through IKK pathway. In PIWIL2 overexpressed KYSE150 cells co-transfected with Ad- mRFP-GFP-LC3 vectors, both yellow (autophagosomes) and red (autolysosomes) puncta were significantly increased. Treatment with 10 μM BAY11-7082 can effectively decrease both signals. However, the number of both puncta has no significant change in cells co-transfection with p65 shRNA (Fig. 5a). WB results also showed that upregulation of autophagic marker LC3-II, p62, and Beclin-1 by PIWIL2 overexpression can be rescued by treatment with BAY11-7082 (Fig. 5b), but not by p65 knockdown (Fig. 5c), suggesting that PIWIL2 induces autophagy in an IKK-dependent but NF-κB-independent manner. The selectiveness of BAY11-7082 in inhibiting IKK-induced autophagy was shown in Fig. S4.

To specify the mechanism of PIWIL2 regulating autophagy, WB assay was performed to determine the effect of PIWIL2 on the classical mTOR pathway. The results showed that PIWIL2 overexpression can deactivate mTORC1 and subsequently increase the phosphorylation level of ULK1. On the contrary, PIWIL2 knockdown can increase phosphorylation level of mTORC1 and suppress ULK phosphorylation (Fig. 5d, e). Treatment with 100 nM rapamycin can inhibit the activity of mTORC1 and promote autophagy stimulated by PIWIL2 knockdown in ESCC cells, suggesting that PIWIL2 induces autophagy by suppressing mTORC1 pathway (Fig. 5f). In addition, inhibition of mTORC1 by PIWIL2 can be rescued by treatment of BAY11-7082 (Fig. 5g).

Previous study suggested that IKKβ promotes mTOR pathway by deactivating TSC1, which suppresses Rheb and subsequently deactivates mTORC1 [16]. Here, we showed that overexpression of PIWIL2 can block the interaction between IKKβ and TSC1 in KYSE150 cells transfected with varied dose of *PIWIL2* expression vector. With higher expression of PIWIL2, more IKKβ can be pulled down by anti-PIWIL2, and less IKKβ can be pulled down by anti-TSC1 (Fig. 5h). In addition, TnT system was employed to express PIWIL2, IKKβ, and TSC1 in a cell-free condition. Co-IP analysis showed that PIWIL2 can directly bind to IKKβ, and block the interaction between IKKβ and TSC1 (Fig. 5i). In accord to this finding, overexpression of Rheb can rescue the mTORC1 pathway suppressed by PIWIL2 overexpression (Fig. 5j).

Interestingly, co-transfected with vectors encoding a kinase-dead variant of IKKβ (K44A) can rescue mTORC1 activity in control KYSE150 cells, but not in PIWIL2 overexpressed cells, suggesting that IKKβ may activate mTORC1 in a kinase-independent manner (Fig. 5k).

### PIWIL2 promotes ESCC growth IKK-dependently in a xenograft mouse model

To examine the role of PIWIL2 in ESCC in vivo, PIWIL2 knockdown stable cells were subcutaneously injected into 6-week-old nude mice, and wildtype KYSE150 cells were used as control. Two weeks after implantation, the mice were random divided into different groups and intratumoral injected with IKK inhibitor BAY11-7082 (5 mg/kg) every 3 days. Our results revealed that mice injected with PIWIL2 knockdown stable cells showed dramatically decreased tumor volume compared to those injected with wildtype cells (376.5 ± 213.5 mm^3^ vs 1665.9 ± 275.4 mm^3^, *p* = 0.008).

**Fig. 6:**
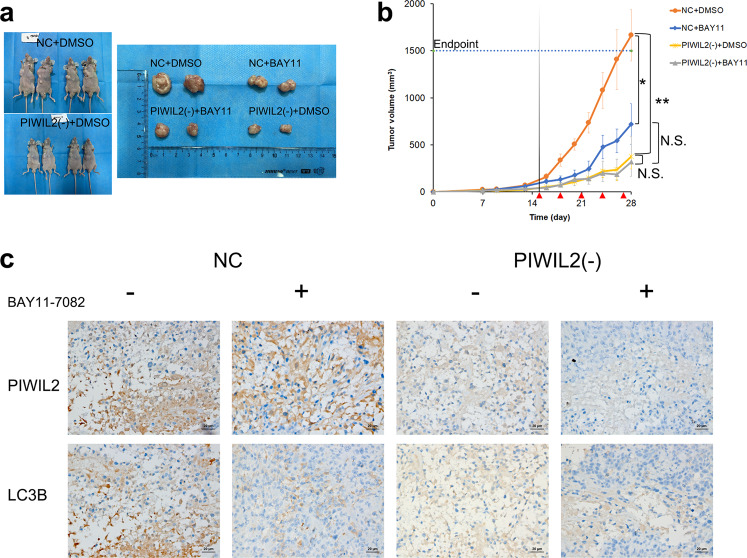
PIWIL2 promotes tumor growth in the KYSE150 xenograft model. **a**–**c** BALB/c nude mice were implanted with PIWIL2 knockdown stable cells [PIWIL2(−)] or wildtype KYSE150 cells (NC), then intratumoral injected with 5 mg/kg BAY11-7082 or vehicle control (DMSO); *n* = 6 per group. **a** Left, representative photos of two mice from each of the groups injected with DMSO. Right, representative photos of two tumor tissues from each group. **b** Tumor growth rate was measured by Volume (mm^3^) = (Length × Width^2^)/2. Red arrows, intratumoral injection with 5 mg/kg BAY11-7082. N.S., no significance; *p < 0.05; **p < 0.01. **c** Representative images of immunohistochemical analysis of PIWIL2 and LC3B for tissues of xenograft tumors. Scale bar, 20 µm.

Notably, treatment with BAY11-7082 reduced average tumor size in mice implanted with wildtype KYSE150 to 721.2 ± 217.0 mm^3^, showing significant difference from the vehicle controls (*p* = 0.014). But no significant difference in tumor volume can be observed between the treatment group and the control group implanted with PIWIL2 knockdown stable cells (316.5 ± 71.4 mm^3^ vs 376.5 ± 213.5 mm^3^, *p* = 0.780). Also, treatment of BAY11-7082 can abolish the significant difference between PIWIL2 knockdown stable cells and wildtype cells (*p* = 0.183). These results indicated that PIWIL2 promotes the growth of ESCC in vivo in an IKK-dependent manner (Fig. 6a, b). Additionally, IHC analysis showed that LC3B staining is downregulated in tumors derived from PIWIL2 knockdown stable cells, compared with wildtype KYSE150 cells. And injection of BAY11-7082 can abolish the difference (Fig. 6c).

## Discussion

Previous studies have shown that radiotherapy and chemotherapy can induce autophagy in ESCC cells and inhibition of autophagy can enhance the sensitivity to radiotherapy and chemotherapy of ESCC, suggesting that autophagy may be a potent target for ESCC therapy [5, 7, 8, 27–29]. However, while autophagy as well as apoptosis suppress is of great importance for the survival of tumor cells, the underlying mechanism of regulating autophagy and apoptosis in ESCC remains largely unknown [30, 31].

Our present research showed that PIWIL2 is highly expressed in both solid tumor from ESCC patients and established ESCC cell lines (Fig. 1a–c). The high-grade expression of PIWIL2 correlates with high T-stage and is an independent factor of poor prognosis in ESCC patients (Fig. 1d and Table 2). In ESCC cell lines, overexpressing of PIWIL2 significantly promotes cell proliferation, inhibits apoptosis, and activates autophagy (Fig. 2), revealing its potent roles in ESCC. Interestingly, our data showed that PIWIL2 can directly interact with IKK, promoting its expression and phosphorylation level (Fig. 3). PIWIL2 induced activation of IKK then induces phosphorylation of IκB and releasing of NF-κB for nuclear translocation and apoptosis inhibition (Fig. 4), suggesting that PIWIL2 can repress apoptosis in ESCC cells by activating the classic IKK/IκB/NF-κB pathway.

Notably, while previous study suggested that NF-κB pathway may play roles in regulating autophagy [32], our present work revealed that PIWIL2 can activate autophagy in an IKK-dependent but NF-κB-independent manner (Fig. 5a–c). Further study suggested that PIWIL2 can block the interaction of IKKβ with TSC1. Released TSC1 further deactivate mTORC1, promotes ULK1 phosphorylation, and initiates autophagy (Fig. 5d–k), suggesting that the competitive binding of PIWIL2 and TSC1 to IKK may result to initiation of autophagy by inhibiting the mTORC1 pathway. In accord to these findings, the mouse xenograft model showed that PIWIL2 can promote ESCC growth in an IKK-dependent manner (Fig. 6).

**Fig. 7:**
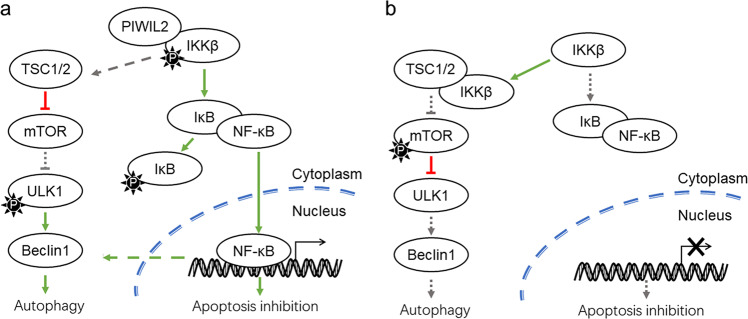
Working model of PIWIL2 regulating apoptosis and autophagy in ESCC cells. **a** In cells with PIWIL2 expression. **b** In PIWIL2 absent cells. P, phosphate.

In summary, our present study proposes a hypothetical mechanism that PIWIL2 regulates apoptosis and autophagy via IKK pathway in ESCC. Firstly, PIWIL2 can bind to IKK and promote its phosphorylation, leading to phosphorylation of IκB and subsequently nuclear translocation of NF-κB for apoptosis inhibition. Secondly, PIWIL2 competitively inhibits binding of IKKβ to TSC1, and thus deactivate mTORC1 which suppress ULK1 phosphorylation and initiation of autophagy (Fig. 7).

Therefore, by suppressing apoptosis and inducing autophagy, PIWIL2 plays roles in the survival of tumor cells and associates with poor prognosis in ESCC patients. Taken together, our work first revealed the molecular mechanism that PIWIL2 regulates apoptosis and autophagy in ESCC, providing a novel insight into roles of PIWIL2 in tumorigenesis.

## Materials and methods

### Patients and tissue specimens

A total of 109 cases of matched ESCC and paratumor tissue samples were collected from patients who were diagnosed with primary ESCC and received radical esophageal surgery without preoperative chemoradiotherapy from 2009 to 2011 at West China Hospital, Sichuan University (Chengdu, China). Patients were classified according to TNM staging (American Joint Committee on Cancer, 8th edition), and well documented with complete follow-ups for 5 years or until death. The samples were obtained with informed consent according to an established protocol approved by the Ethics Committee of West China Hospital, Sichuan University.

### Tissue microarrays analysis and immunohistochemistry

TMA blocks were constructed using paraffin-embedded, formalin-fixed tissues from 109 patients. The slide was deparaffinized and rehydrated, followed by treatment with 3% H_2_O_2_ to block endogenous peroxidases. After incubation of primary antibody, and secondary antibody, the sections were semiquantitatively scored for the PIWIL2 staining patterns as follows: the staining intensity was quantified as 0 (negative), 1 (weak), 2 (intermediate), or 3 (strong). The percentage of immunoreactive tumor cells was scored as follows: 0 (0–5%), 1 (6–25%), 2 (26–50%), 3 (51–75%), or 4 (76–100%). The final immunoreactive score was obtained by multiplying the percentage and the intensity values (range 0–12), and the samples were divided into low-grade PIWIL2 expression group (score <6) and high-grade PIWIL2 expression group (score 6–12).

### Cell lines and culture conditions

Human ESCC cell line KYSE150 was purchased from Shanghai Zhongqiaoxinzhou Biotechnology (Shanghai, China). Cell lines of KYSE510, TE-1, Eca-109, KYSE180, and Het-1A were maintained in the laboratory of thoracic surgery of West China Hospital, Sichuan University. All ESCC cells were cultured in RPMI-1640 (Gibco, USA), and Het-1A cells was cultured in DMEM (Gibco, USA), supplemented with 10% fetal bovine serum, 100 U/ml penicillin and 100 µg/ml streptomycin, in an incubator with 5% CO_2_ at 37 °C. The transfection was performed with jetPRIME^™^ (Polyplus-transfection, France) according to the manufacturer’s instruction. For construction of PIWIL2 knockdown stable cells derived from KYSE150, transfected cells were selected at 72 h after transfection by treatment of 1 mg/ml G418 for 3–4 weeks. The stable cells lines were further identified by detecting PIWIL2 expression by Western blotting. To isolate the cytoplasmic component from the nuclear one, cells were treated with the cytosolic (C)/nuclear (N) fractionation extraction kit (Beyotime, China) according to the manufacturer’s instructions.

### Plasmids and antibodies

CDS encoding PIWIL2 and p65 was synthesized and inserted into pcDNA3.1 and pEGFP, respectively. shRNA against PIWIL2 and p65 were synthesized by TSINGKE Biological Technology company (Beijing, China) and the target sequences of these shRNA were as follows:

Human PIWIL2 shRNA (shPIWIL2-1): 5′-CGG ATT GAG GAG AAA CGT AAA CTC-3′

Human PIWIL2 shRNA (shPIWIL2-2): 5′-CTA TGA GAT TCC TCA ACT ACA GAA G-3′

Human p65 shRNA (shp65): 5′ -CGG ATT GAG GAG AAA CGT AAA CTC-3′

Anti-PIWIL2, anti-p62, anti-Beclin-1, and anti-LaminB1 were purchased from Santa Cruz (USA). Anti-IKKα, anti-IKKβ, anti-p-IKKα/β(Ser176/180), anti-IκBα, anti-p-IκBα(Ser32/36), anti-p65, anti-p-mTOR(Ser2448), anti-p-ULK1(Ser555), anti-p-4E-BP1(Thr37/46), anti-p-P70S6(Thr389), and anti-TSC1 were purchased from Cell signaling technology (USA). Anti-LC3B was purchased from Novus Biologicals (USA). Anti-mTOR and anti-Bcl-2 were purchased from Proteintech Group (USA). Anti-GAPDH was purchased from Epitomics (USA).

### Real-time PCR

Quantitative PCR was performed using an iCycler IQ^™^ system (BIO-RAD, USA) with a denaturation step at 94 °C for 10 min, followed by 40 cycles of denaturation at 94 °C for 20 s, annealing at 60 °C for 30 s, and extension at 72 °C for 40 s using the SYBR Green Master Mix (Takara, Japan).

### Co-immunoprecipitation and western blotting

For co-immunoprecipitation and western blotting, cells were lysed after transfecting with the designated plasmids in universal protein extraction buffers (Bioteke, China) containing protease inhibitor cocktail (Roche, Switzerland). Extracted proteins were immunoprecipitated with special antibody and protein A + G agarose beads (Beyotime, China). Bound proteins were separated using sodium dodecyl sulfate (SDS)–polyacrylamide gel electrophoresis, transferred to polyvinylidene difluoride membranes (Merk-Millipore, Germany), and detected with specific appropriate primary antibodies and horseradish peroxidase-conjugated secondary antibodies. Specific proteins were visualized using Immobilon^™^ chemiluminescence western blotting detection system (Merk-Millipore, Germany).

### Ad-mRFP-GFP-LC3B transfection

Ad-mRFP-GFP-LC3B vectors were transfected according to the manufacturer’s instruction (Hanbio Biotechnology, China), and transfected cells were cultured for 72 h in an environment at 37 °C and 5% CO_2_.

### Confocal and transmission electron imaging

Cells cultured on 12-well chamber slides were fixed in 4% paraformaldehyde for 15 min, treated with 0.2% Triton X-100 in PBS for 3 min and incubated with 1% BSA for 1 h at RT. Following incubation with primary antibodies at 4 °C overnight, the cells were washed three times in PBS and incubated with secondary antibodies for 2 h at RT. DAPI (Sigma-Aldrich, USA) was used to stain the nuclei. Images were visualized by an orthostatic two-photon confocal microscope (Nikon, Japan).

For transmission electron imaging, KYSE150 cells were collected and washed twice with precooled PBS, and then fixed in 2.5% glutaraldehyde for 24 h. Subsequently, the samples were sent to Wuhan Servicebio Technology (Wuhan, China) for transmission electron imaging.

### Cell proliferation, apoptosis, and colony formation assay

The KYSE150 cells were seeded at a density of 1 × 10^3^ per well in 96-well plates. At 12, 24, 36, or 48 h post-transfection, cells were harvested and cell proliferation was analyzed using crystal violet assay, according to the manufacturer’s instructions. For cell apoptosis analysis, cells were treated according to the instructions provided with the Hochest33342/PI apoptosis detection kit (Solarbio, China). And the apoptotic ratios were analyzed by a Coulter Epics XL flow cytometer (Beckman, USA). For colony formation assay, 1 × 10^4^ cells suspended in 0.7% soft agar were plated on the surface of 1.2% soft agar in six-well cell culture plate. After 2-weeks growth, colony was counted under ×100 microscope.

### In vivo tumor xenograft model

BALB/c nude mice (4–6 weeks old) were purchased from the animal facility of West China Hospital (Chengdu, China). Animals were randomly assigned to each group using a random number generator (EXCEL), but experiments thereafter were not blinded. A sample size of six mice per group was used and was calculated to have 90% power (standard deviation 25%, alpha 0.05) to detect an 80% difference in tumor volume, based on G*Power 3.1 [33]. PIWIL2 knockdown stable cells (1 × 10^7^) or wildtype KYSE150 cells as control were suspended in 200 μl culture medium and subcutaneously injected into nude mice. In total, 14 days after implantation, 5 mg/kg BAY11-7082 or vehicle control (DMSO) were intratumoral injected every 3 days, six mice per group. Tumor growth rate was measured every 2 days and the tumor volume was calculated as follows: Volume (mm^3^) = (Length × Width^2^)/2. The animals were euthanized when reached the endpoint tumor size of 1500 mm^3^, or 4 weeks after implantation. The tumors were fixed in 4% paraformaldehyde, sectioned, and subjected to immunohistochemical analysis. All animal experiments were approved by the Animal Ethics Committee of West China Hospital.

### Statistical analysis

Statistical analyses were performed using GraphPad Prism 8.0 software (GraphPad Software, USA). Continuous variables were presented as mean ± standard error of the mean, and the data from two groups were analyzed by student’s *t* test. Survival curves were drawn using Kaplan–Meier and the difference of survival rate among subgroups was compared by Log-rank test. Univariate and multivariate statistical analysis was performed according to the parameters provided by the multivariate Cox proportional hazard model. X-tile 3.6 software (Yale University School of Medicine, New Haven, USA) was used for statistical analysis of the immunohistochemical comprehensive score. *t*-test or Fisher’s accurate test were used for the comparison of composition ratio between groups, and Pearson correlation analysis was used for the correlation test of the two groups of data. Statistical significance was accepted when *p* < 0.05. All experiments were repeated at least three times unless stated otherwise.

## Supplementary information

Supplementary Figure legends

Supplemental information for TMA

Flow cytometry analysis with Hochest33342/PI double staining showed that PIWIL2 significantly suppress the apoptosis of KYSE180 cells.

Binding mode of IKKβ-PIWIL2 protein complexes.

The selectively inhibition of IKK-induced autophagy by BAY11-7082
