# Supplementary material for: PIWIL2 interacting with IKK to regulate autophagy and apoptosis in esophageal squamous cell carcinoma
Source: Cell Death Differ. 2021 Jan 19;28(6):1941–54. doi: 10.1038/s41418-020-00725-4 (PMC8184941; doi:10.1038/s41418-020-00725-4)
Supplement: Supplementary file 1 — Supplementary Figure legends [file 41418_2020_725_MOESM1_ESM.docx]

**Legend for Supplementary Figures**

**Figure S1** (**a**) The expression of PIWIL2 in 109 patients with ESCC was scored by staining intensity (0-3) and area (0-4) of the staining with tissue microarray technology and immunohistochemistry. Representations of intensity quantification: 0 (negative), 1 (weak), 2 (intermediate), and 3 (strong). The final scores of all samples were analyzed using X-tile software. It is showed that the maximum chi-square value is obtained when the score is 4. The scores of all samples were successively divided into the low-expression PIWIL2 group (score 0-4) and the high-expression PIWIL2 group (score 6-12). (**b**) The K-M curve of each clinicopathological index. The Log-rank test indicated that the survival differences of T stage, N stage and TNM stage were statistically significant (p < 0.05).

**Figure S2** Flow cytometry analysis with Hochest33342/PI double staining showed that PIWIL2 significantly suppress the apoptosis of KYSE180 cells.

**Figure S3** Binding mode of IKKβ-PIWIL2 protein complexes. The results of interface analysis showed that Arg53, Ser60, Asp61, Lys73, Tyr75 from PIWIL2 and Glu64, Arg140, Asp145, Phe182 and Leu186 from IKKβ on the interface had close interactions with each other.

**Figure S4** The selectively inhibition of IKK-induced autophagy by BAY11-7082.
