# Supplementary figures and images for: PIWIL2 interacting with IKK to regulate autophagy and apoptosis in esophageal squamous cell carcinoma
Source: Cell Death Differ. 2021 Jan 19;28(6):1941–54. doi: 10.1038/s41418-020-00725-4 (PMC8184941; doi:10.1038/s41418-020-00725-4)

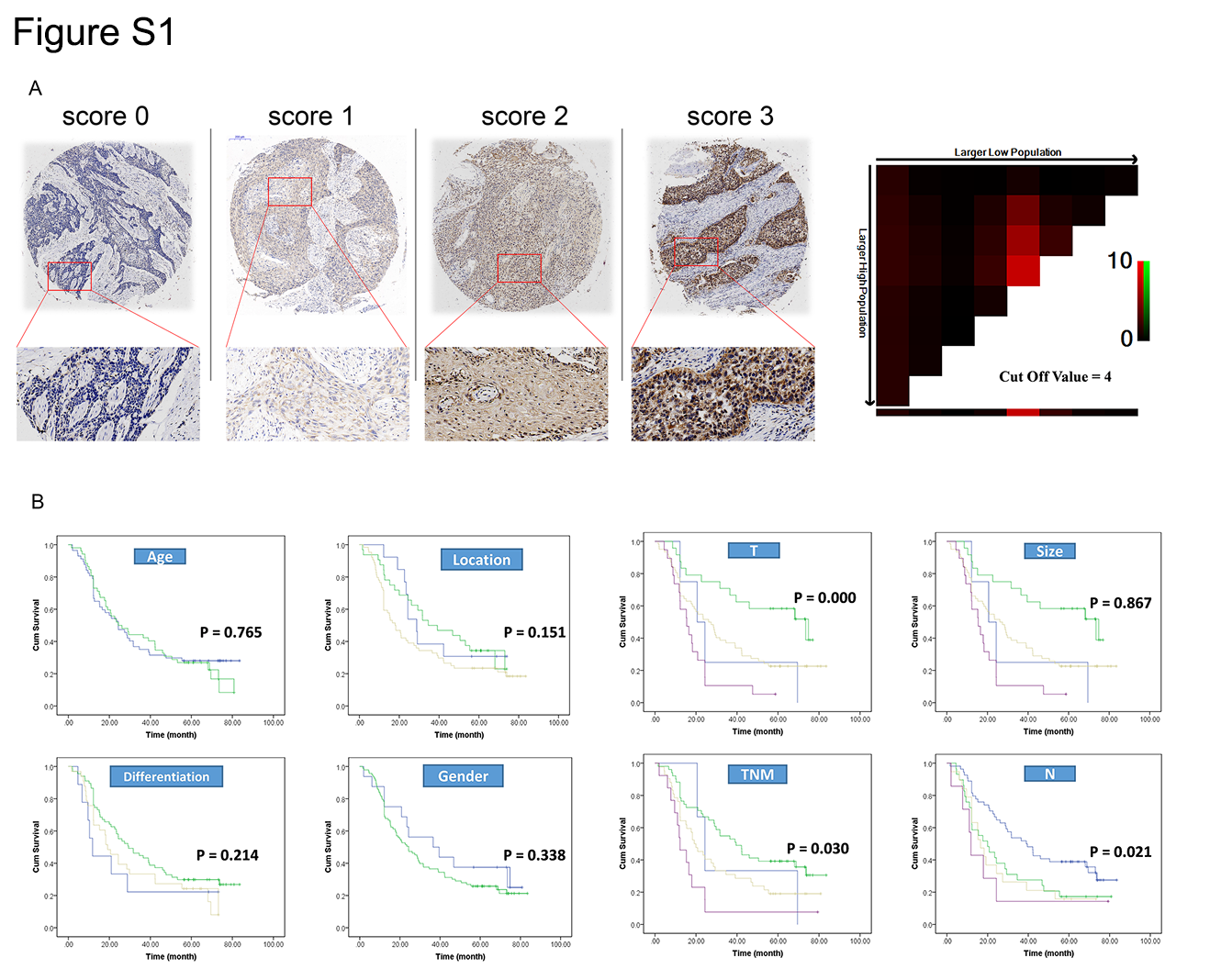

Supplement: Supplementary file 2 — Supplemental information for TMA [file 41418_2020_725_MOESM2_ESM.tif]

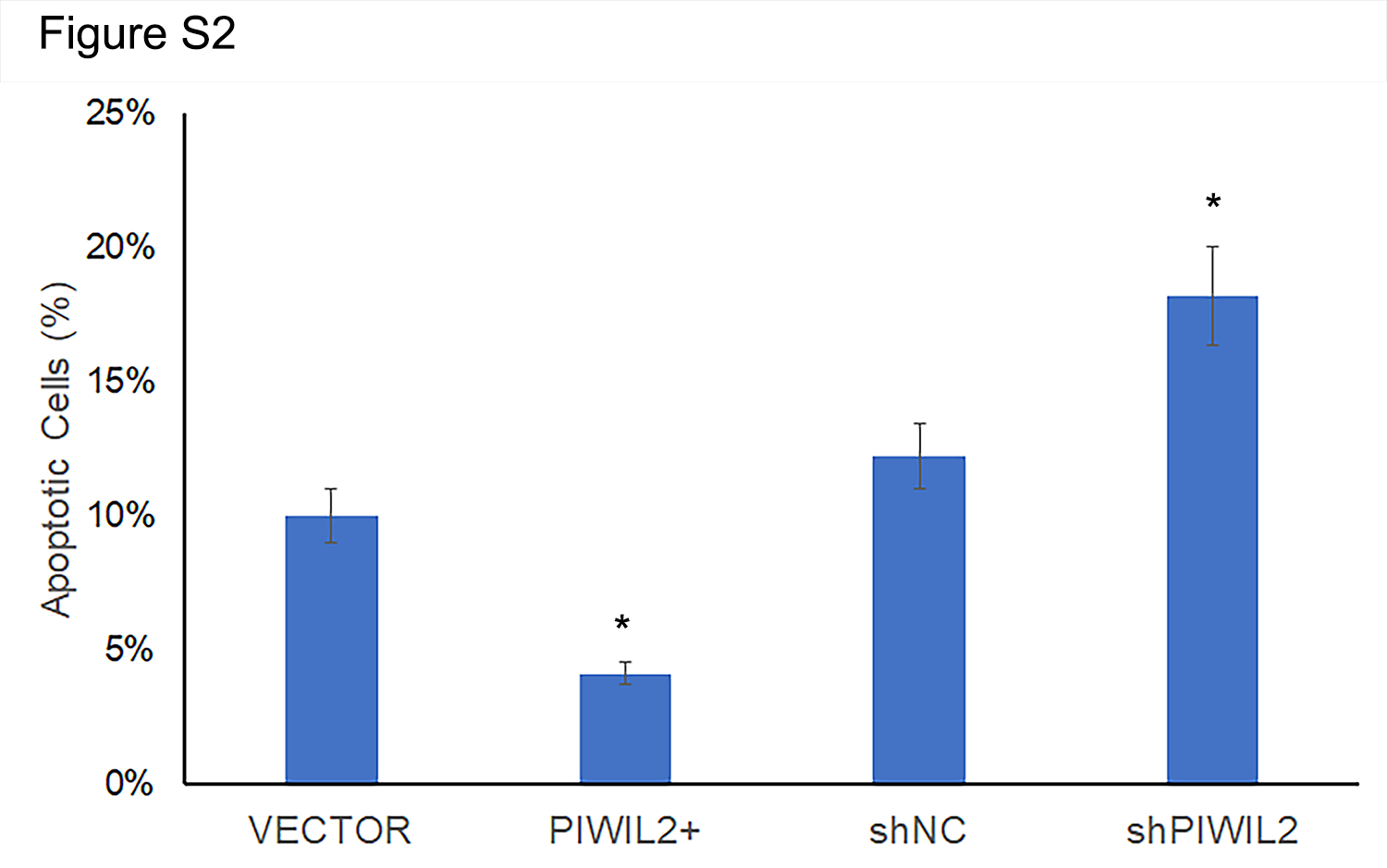

Supplement: Supplementary file 3 — Flow cytometry analysis with Hochest33342/PI double staining showed that PIWIL2 significantly suppress the apoptosis of KYSE180 cells. [file 41418_2020_725_MOESM3_ESM.tif]

Figure S2

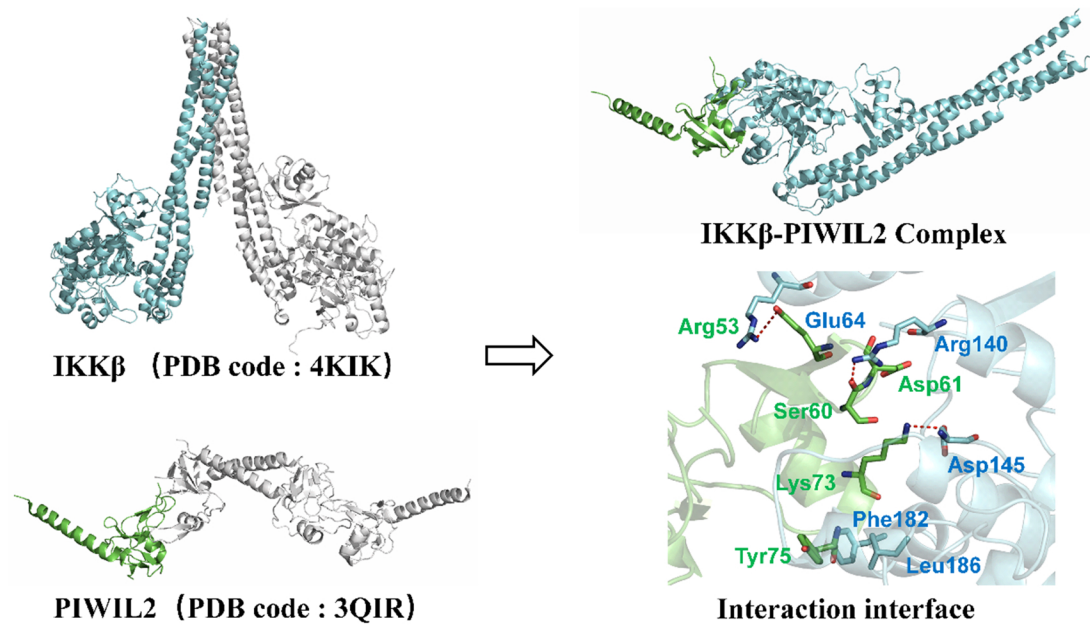

Supplement: Supplementary file 4 — Binding mode of IKKβ-PIWIL2 protein complexes. [file 41418_2020_725_MOESM4_ESM.pdf]

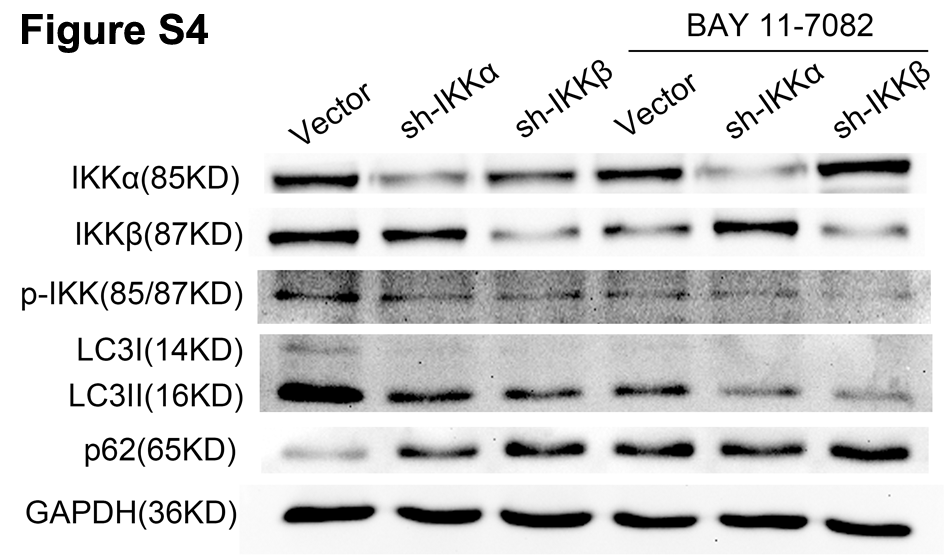

Supplement: Supplementary file 5 — The selectively inhibition of IKK-induced autophagy by BAY11-7082 [file 41418_2020_725_MOESM5_ESM.tif]
